# Serum amyloid A triggers the mosodium urate -mediated mature interleukin-1β production from human synovial fibroblasts

**DOI:** 10.1186/ar3849

**Published:** 2012-05-18

**Authors:** Kiyoshi Migita, Tomohiro Koga, Kenshi Satomura, Masahiro Izumi, Takafumi Torigoshi, Yumi Maeda, Yasumori Izumi, Yuka Jiuchi, Taiichiro Miyashita, Satoshi Yamasaki, Yoshihiro Aiba, Atsumasa Komori, Minoru Nakamura, Satoru Motokawa, Atsushi Kawakami, Tadashi Nakamura, Hiromi Ishibashi

**Affiliations:** 1 Department of Rheumatology, Nagasaki Medical Center, Kubara 2-1001-1, Omura, Nagasaki 856-8562, Japan; 2 Orthopedic Surgery, Nagasaki Medical Center, Kubara 2-1001-1, Omura, Nagasaki 856-8562, Japan; 3 Clinical Research Center, Nagasaki Medical Center, Kubara 2-1001-1, Omura, Nagasaki 856-8562, Japan; 4 Department of Rheumatology, Nagasaki University Hospital, Sakamoto 1-7-1, Nagasaki 852-8201, Japan; 5 Department of Rheumatology, NTT West Japan Hospital, Shinyashiki 1-17 Kumamoto 862-8655, Japan

## Abstract

**Background:**

Monosodium urate (MSU) has been shown to promote inflammasome activation and interleukin-1β (IL-1β) secretion in monocyte/macrophages, but the cellular pathway and nod-like receptor family, pyrin domain containing 3 (NLRP3) inflammasome activation in synovial tissues, remain elusive. In this study, we investigated the effects of MSU on synovial fibroblasts to elucidate the process of MSU-mediated synovial inflammation.

**Methods:**

Human synovial fibroblasts were stimulated with MSU in the presence or absence of serum amyloid A (SAA). The cellular supernatants were analyzed by immunoblotting using anti-IL-1β or anti-caspase-1 antibodies. IL-1β or NLRP3 mRNA expressions were analyzed by real-time PCR or reverse transcription-PCR (RT-PCR) method.

**Results:**

Neither SAA nor MSU stimulation resulted in IL-1β or interleukin-1α (IL-1α) secretions and pro-IL-1β processing in synovial fibroblasts. However, in SAA-primed synovial fibroblasts, MSU stimulation resulted in the activation of caspase-1 and production of active IL-1β and IL-1α. The effect of SAA on IL-1β induction was impaired in cells by silencing NLRP3 using siRNA or treating with caspase-1 inhibitor. In addition, SAA induced the secretion of cathepsin B and NLRP3 mRNA expression in synovial fibroblasts.

**Conclusions:**

Our data demonstrate that exposure of human synovial fibroblasts to SAA promotes MSU-mediated caspase-1 activation and IL-1β secretion in the absence of microbial stimulation. These findings provide insight into the molecular processes underlying the synovial inflammatory condition of gout.

## Introduction

Gout is a paradigm for acute sterile inflammation that is triggered by interactions between monosodium urate (MSU) crystals and inflammatory cells in the joint connective tissues [1]. Interleukin-1β (IL-1β) has been identified as a pivotal cytokine in gout and MSU crystal-induced inflammation [2]. IL-1β is induced as an inactive pro-molecule by immune cells, such as macrophages and monocytes, and then cleaved into the active p17 form of IL-1 by caspase-1 [3,4]. Tschopp *et al*. demonstrated that MSU is capable of activating the NLRP3 inflammasome to process and secrete active IL-1β [5]. These findings suggest that macrophages can recognize MSU as danger-associated molecular patterns (DAMPs) in the damaged tissues and release proinflammatory IL-1β [6]. Upon activation, NLRP3 binds to the ASC, which in turn recruits procaspase-1 for activation. Activated caspase-1 cleaves pro-IL-1β to form the mature IL-1β [7].

*In vitro *experiments have shown that triggering of the inflammasome to process IL-1β is a multistep process. Lipopolysaccharide, which belongs to pathogen-associated molecular patterns (PAMPs), had been shown to induce IL-1β from human synovial macrophages [8]. In the absence of a first signal that induces pro-IL-1β, such as lipopolysaccharide, monocyte/macrophages do not spontaneously secrete mature IL-1β when stimulated with NLRP3 ligands [9,10]. Ii proposed that the first signal modulates the threshold of NLRP3 and the second signal activates NLRP3 inflammasome and causes subsequent caspase-1 activation and IL-1β processing [11,12]. Recent investigations demonstrated that IL-1β and IL-1 receptor are key players in MSU-mediated acute inflammation [13,14]. However, the steps that associate cellular activity with MSU crystals that induce inflammasome activation in gouty arthritis are not completely understood.

Serum amyloid A (SAA) is an acute-phase protein present in serum. SAA is also known to possess proinflammatory properties and to mediate inflammatory disease pathogenesis [15,16]. It has recently been demonstrated that β-amyloid fibrils in Alzheimer's disease signal through the NLRP3 inflammasome and drive caspase-1-dependent cleavage of IL-1β [17]. Furthermore, SAA has been shown to induce the expression of IL-1β and activate the NLRP3 inflammasome *via *a cathepsin B- and P2X_7_-dependent manner [18]. In this study, we investigated the MSU-mediated NLRP3 activation process using synovial fibroblasts isolated from human synovium and adjuvant activity induced by SAA.

## Materials and methods

### Reagents

Recombinant human SAA was purchased from Peprotech (Rocky Hills, NJ, USA). According to the manufacturer, the endotoxin level of the product is 0.1 ng/mg protein. MSU crystals were purchased from Alexis (Lausen, Switzerland). Polyclonal anti-IL-1β, pro-IL-1β and anti-cleaved caspase-1 (D57A2) antibodies were purchased from Cell Signaling Technology (Beverly, MA, USA). Anti-caspase-1 polyclonal antibodies (sc-622) were purchased from Santa Cruz Biotechnology (Santa Cruz, CA, USA). Anti-NLRP3 antibodies were obtained from Abcam (Cambridge, UK). Anti-cathepsin B antibodies and caspase-1 inhibitor (z-YVAD-FMK) were obtained from Calbiochem (San Diego, CA, USA)

### Preparation of synovial fibroblasts

Synovial tissues were obtained from patients with rheumatoid arthritis at the time of total joint replacement. Synovial fibroblasts were isolated from the synovial tissues by enzymatic digestion. The study was approved by the Ethics Committees Nagasaki Medical Center and informed consent was obtained from each of the individuals. Synovial fibroblasts were used from passages 4 through 6 during which time they are a homogeneous population of cells (<1% CD 45 positive).

### Measurement of cytokine secretion and immunoblot analysis

Synovial fibrablasts (5 × 10^4^) were seeded in 24-well plates containing RPMI1640 supplemented with 10% heat-inactivated FBS and stimulated with MSU for 24 hours. In some experiments, synovial fibrablasts were pre-treated with SAA for 12 hours before stimulation. Cell-free supernatants were collected by centrifugation at 400 g for five minutes and assayed for IL-1β or IL-1α with enzyme-linked immunosorbent assay (ELISA) kits (R&D Systems, Minneapolis, MN, USA) without the steps for concentrations or precipitations. The same supernatants were also subjected to 12% SDS-PAGE, followed by immunoblotting with Abs for human IL-1β (dilution 1:400), caspase-1 (dilution 1:500), and cathepsin B (dilution 1:500) with an ECL Western blotting kit (Amersham, Little Chalfont, UK). Endotoxin was measured by chromogenic limulus test (Toxicolor LS-50M Kit, SEIKAGAKU CORPORATION, Tokyo, Japan).

### Small interfering RNA experiments

Synovial fibroblasts were transfected with 100 nM non-targeting control small interfering RNA (siRNA; AllStars Negative Control siRNA; Qiagen, Hilden, Germany) or with 50 nM two NLRP3 siRNAs (CIAS1_6 and CIAS1_9; Qiagen), combined with the HiPerFect Transfection Reagent (Qiagen) under serum-free condition, as instructed by the manufacturer. The medium was subsequently replaced, pretreated with SAA for 12 h and stimulated with another 24 h with MSU with medium containing 10% FBS. The cell-culture medium was collected for IL-1β ELISA analysis. In some experiments cells were harvested for total RNA purification after SAA pretreatment and analyzed by semi-quantitative RT-PCR (NLRP3), as described below.

### Reverse transcription-polymerase chain reaction (RT-PCR)

Total RNA was extracted from synovial fibroblasts using the RNeasy total RNA isolation protocol (Qiagen, Crauley, UK) according to the manufacturer's protocol. First-strand cDNA was synthesized from 1 μg of total cellular RNA using an RNA PCR kit (Takara Bio Inc., Otsu, Japan) with random primers. Thereafter, cDNA was amplified using specific primers respectively. The specific primers used were as follows:

NLRP3: forward primer 5'- AAAGAGATGAGCCGAAGTGGG -3' reverse primer 5'- TCAATGCTGTCTTCCTGGCA -3' β-actin; forward primer 5'-GTGGGGCGCCCCAGGCACCA-3' reverse primer 5'-CTCCTTAATGTCACGCACGATTTC-3'.

The product sizes were 79 bp for NLRP3 and 234 bp for β-actin. The thermocycling conditions (35 cycles) 94°C for 60 s and 62°C for 60 s, and 72°C for 60 s.

The amplification of the IL-1-β transcripts was also accomplished on a Light Cycler (Roche Diagnostics, Mannheim, Germany) using specific primers. The housekeeping gene fragment of glyceraldehydes-3-phosphates dehydrogenase (GAPDH) was used for verification of equal loading.

### Cell lysis and immunoblot

Synovial fibroblasts were stimulated with SAA with the indicated concentrations of SAA for 24 h. Cells were washed by ice-cold PBS and lysed with a lysis buffer (1% Nonidet P 40, 50 mM Tris, pH 7.5, 100 mM NaCl, 50 mM NaF, 5 mM EDTA , 20 mM β-glycerophosphate, 1.0 mM sodium orthovanadate, 10 μg/mL aprotinin and 10 μg/mL leupeptin) for 20 minutes at 4°C. Insoluble material was removed by centrifugation at 15,000 × g for 15 minutes at 4°C. The supernatant was saved and the protein concentration was determined using the Bio-Rad protein assay kit (Bio Rad, Hercules, CA, USA). An identical amount of protein (50 μg) for each lysate was subjected to 10% SDS-polyacrylamide gel electrophoresis, and then transferred to a nitrocellulose membrane. Immunoblot analysis using anti-NLRP3, pro-IL-β and β-acitin antibodies was performed with an ECL Western blotting kit (GE Healthcare, BUCKS, UK). In brief, the membrane was probed with primary antibodies and washed and incubated with donkey anti-rabbit secondary antibody conjugated with horseradish peroxidase (1:10,000 diluation; GE Healthcare). After being washed, the membrane was reacted with an ECL advance Western blot detection kit (GE Healthcare). Protein bands were visualized using a lumino-image analyzer (LAS3000; Fujifilm, Toyo, Japan).

### Statistical analysis

Differences between groups were examined for statistical significance using Wilcoxon-Mann-Whitney U test. *P-*values less than 0.05 were considered statistically significance.

## Results

### SAA priming induces mature IL-1β secretion from MSU-treated synoval fibroblasts

**Figure 1:**
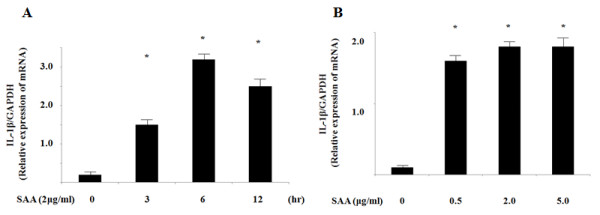
**SAA induces the transcription of pro-IL-1β in human synovial fibroblasts**. **A**. Synovial fibroblasts were incubated with SAA (2 μg/ml) for the indicated periods. The cells were harvested and analyzed for pro-IL-1β mRNA levels by real-time PCR. **B**. Synovial fibroblasts were incubated with the indicated concentrations of SAA for 6 h. The cells were harvested and analyzed for IL-1β and GAPDH mRNA levels by real-time PCR. Values represent the mean ± SD of three independent experiments.*P <0.001 compared to SAA-untreated synovial fibroblasts.

**Figure 2:**
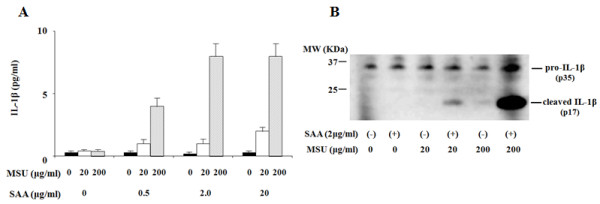
**MSU induces IL-1β synthesis from SAA-primed synovial fibroblasts**. **A**. Synovial fibroblasts were pretreated or untreated with the indicated concentrations of SAA for 12 h. After pretreatment, the cells were stimulated with the indicated concentrations of MSU for 24 h and supernatants were analyzed for IL-1β production by ELISA. Values represent the mean ± SD of three independent experiments. **B**. Synovial fibroblasts were pretreated or untreated with the indicated concentrations of SAA for 12 h. After pretreatment, the cells were stimulated with the indicated concentrations of MSU for 24 h and supernatants were analyzed by immunoblot for the presence of mature IL-1β. Three experiments were performed using different synovial fibroblasts and a representative result is shown.

SAA has been shown to induce the expression of various proinflammatory cytokines in inflammatory cells. First, we examined whether SAA induces IL-1β secretion from synovial fibroblasts. As shown in Figure 1, SAA is a potent inducer of pro-IL-1β mRNA expression in synovial fibroblasts. However, SAA alone did not induce the secretion of IL-1β, suggesting that SAA alone is not able to provide the signal for the proteolytic cleavage of pro-IL-1β and secretion of mature IL-1β (Figure 2A). In contrast, priming of synovial fibroblasts with SAA resulted in the induction of IL-1β secretion when these cells were subsequently stimulated with MSU (Figure 2A). Immunoblot analysis also indicated that in addition to pro-IL-1β (31 kDa), cleaved mature IL-1β (17 kDa) was also induced by MSU in SAA-primed synovial fibroblasts (Figure 2B).

### Endotoxin contamination dose not contribute to the IL-1β induction by SAA/MSA

**Figure 3:**
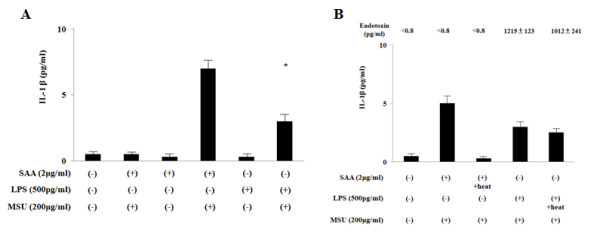
**Endotoxin contaminution dose not contribute to IL-1β induction by SAA/MSU**. **A**. Synovial fibroblasts were pre-treated with SAA (1 μg/ml) or LPS (500 pg/ml) for 12 h. Cells were stimulated with MSU (200 μg/ml) for 24 h. Supernatants were analyzed for IL-1β production by ELISA. Values represent the mean ± SD of two independent experiments. *P *<0.01 compared to SAA-untreated synovial fibroblasts. **B**. Synovial fibroblasts were pre-treated with SAA (1 μg/ml) or LPS (500 pg/ml), which were exposed to 100°C (heat) in a water bath for 30 minutes, for 12 h. Cells were stimulated with MSU (200 μg/ml) for 24 h. Supernatants were analyzed for IL-1β production by ELISA. The endotoxin levels were also measured by limulus test using the same supernatants. Values represent the mean ± SD of two independent experiments.

We next determined whether the induction of IL-1β is a direct effect of SAA or results from contaminating LPS in the SAA preparation. LPS priming induce the IL-1β secretion following MSU stimulation from synovial fibroblasts; however, its IL-1β-inducing ability was lower compared to those of SAA-priming (Figure 3A). Given that most proteins are heat-labile, whereas LPS is heat-resistant, we examined the ability of heat-treated SAA (1 μg/ml) and LPS (500 pg/ml) to stimulate IL-1β secretion from synovial fibroblasts. As shown in Figure 3B, after heating 100°C for 30 minutes, LPS retained its ability to induced MSU-stimulated IL-1β production. In contrast, SAA exposed to 100°C for 30 minutes could not stimulate IL-1β secretion completely as described previously [19]. The endotoxin detected by limulus test was extremely high in LPS-primed synovial fibroblasts-conditioned media, whereas, endotoxin was not detected SAA-primed synovial fibroblast-conditioned media (Figure 3B). These results indicate that the trace amount of LPS in the SAA preparation cannot account for the IL-1β secretion from SAA/MSU-stimulated synovial fibroblasts.

### SAA/MSU-induced IL-1β processing is dependent on caspase-1

**Figure 4:**
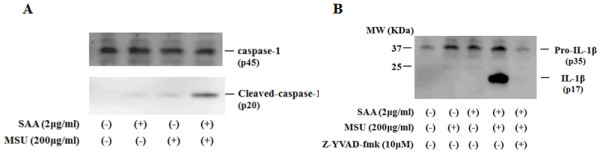
**SAA/MSU-induced IL-1β processing is dependent on caspase-1**. **A**. Synovial fibroblasts were untreated or pretreated with SAA (2 μg/ml) for 12 h. Cells were stimulated with the indicated concentrations of MSU for 24 h. Supernatants were analyzed by immunoblot for the presence of procaspase-1 (p45) and cleaved caspase (p20). Three experiments were performed using different synovial fibroblasts and a representative result is shown. **B**. Synovial fibroblasts were untreated or pretreated with SAA (2 μg/ml) for 12 h. Cells were stimulated with MSU in the presence or absence of z-YVAD-fmk (10 μM) for 24 h. Supernatants were analyzed by immunoblot analysis for the presence of mature IL-1β. Two experiments were performed using different synovial fibroblasts and a representative result is shown.

Previous investigations demonstrated that the inflammatory caspases are cleaved and released along with active IL-1β after activation of the inflammasome [20]. Consistent with these findings, SAA/MSU stimulation activated caspase-1 in synovial fibroblasts (Figure 4A). The caspase-1 dependency for the pro-IL-1β processing was confirmed by addition of the caspase-1 inhibitor z-YVAD-fmk, which completely blocked SAA/MSU-induced IL-1β processing (Figure 4B).

### SAA induces NLRP3 expression and cathepsin B release in synovial fibroblasts

**Figure 5:**
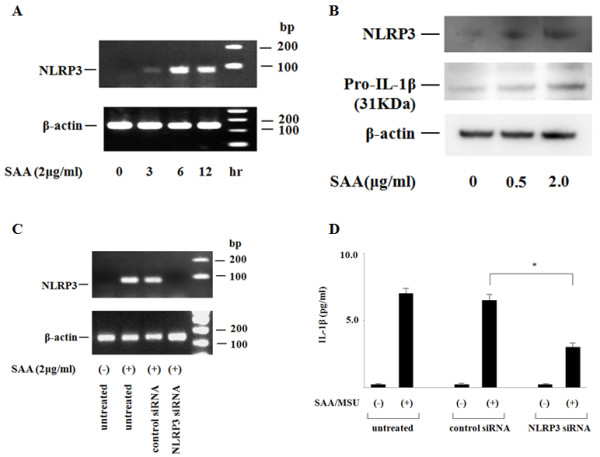
**SAA induces NLRP3 expression**. **A**. Synovial fibroblasts were stimulated with SAA (2 μg/ml) for 12 h. The cells were analyzed for *NLRP3 *mRNA by RT-PCR. Three experiments were performed using different synovial fibroblasts and a representative result is shown. **B**. Synovial fibroblasts were stimulated with the indicated concentrations of SAA for 24 h. Cellular lysates were subjected to Western blotting using specific antibodies against NLRP3, pro-IL-1β and β-actin (internal control). Three experiments were performed and a representative result is shown. **C**. Synovial fibroblasts were transfected with non-targeting control siRNA or NLRP3 siRNA for 22 h and subsequently stimulated with SAA (2 μg/ml) for 12 h. The cells were analyzed for *NLRP3 *mRNA by RT-PCR. Three experiments were performed using different synovial fibroblasts and a representative result is shown. **D**. The transfected cells as described in C were pretreated with SAA (2 μg/ml) for 12 h and subsequently stimulated with MSU (200 μg/ml) for 24 h. Cell-free culture supernatants were analyzed for IL-1β by ELISA. Values represent the mean ± SD of three independent experiments. *P <0.01 compared to control siRNA transfected synovial fibroblasts.

**Figure 6:**
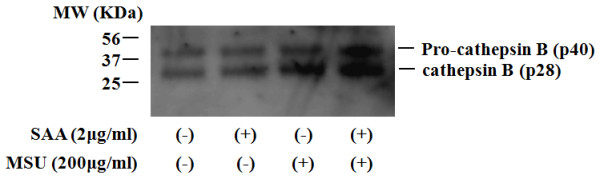
**SAA induces the secretion of cathepsin B from synovial fibroblasts**. Synovial fibroblasts were untreated or pretreated with SAA (2 μg/ml) for 12 h and subsequently stimulated with of MSU (200 μg/ml) for 24 h. Supernatants were analyzed by immunoblot analysis for presence of cathepsin B. Three experiments were performed using different synovial fibroblasts and a representative result is shown.

The induction of NLRP3 expression is important for inflammasome activation [21]. Therefore, we examined the effects of SAA on NLRP3 expression in synovial fibroblasts. As shown in Figure 5A, a rapid increase in NLRP3 mRNA expression was observed in SAA-stimulated synovial fibroblasts. Also, we analyzed the protein expressions of pro-IL-1β and NLRP3 using the lysates of SAA-stimulated synovial fibroblasts. Immunoblot analysis revealed the protein expressions of pro-IL-1β and NLRP3 were increased by SAA stimulation (Figure 5B). To elucidate the role of NLRP3 in SAA/MSU-induced IL-1β induction, NLRP3 was silenced in synovial fibroblasts using a combination of two small interfering RNAs (siRNA). The siRNA treatments prevented SAA-induced *NLRP3 *mRNA expression (Figure 5C). Silencing the *NLRP3 *gene reduced SAA/MSU-induced IL-1β secretion, while no inhibition of IL-1β secretion was observed in synovial fibroblasts transfected with negative control siRNA (Figure 5D). Inflammasome activation has been associated with the release of cathepsin B from the cells [22,23]. Therefore, we examined the cell-free culture media for the presence of cathepsin B. As shown in Figure 6, significant secretion of cathepsin B was observed in SAA/MSU-stimulated synovial fibroblasts, as well as SAA-primed synovial fibroblasts.

### SAA priming induces IL-1a secretion from MSU-treated synoval fibroblasts

**Figure 7:**
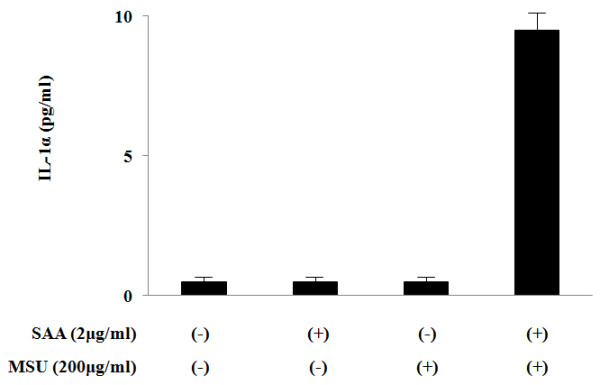
**MSU induces IL-1α synthesis from SAA-primed synovial fibroblasts**. Synovial fibroblasts were pretreated or untreated with the indicated concentrations of SAA for 12 h. After pretreatment, the cells were stimulated with the indicated concentrations of MSU for 24 h and supernatants were analyzed for IL-1*a *production by ELISA. Values represent the mean ± SD of three independent experiments.

More recently, IL-1α secretion was demonstrated to be required the presence of IL-1β, in which IL-1β directly binds IL-1α as a shuttle [24]. Therefore, we examined whether SAA/MSU stimulation induces IL-1α secretion from rheumatoid synovial fibroblasts. Although neither SAA nor MSU induces IL-1α secretion, SAA priming induces the IL-1α secretion from MSU-stimulated synovial fibroblasts parallel to IL-1β secretion (Figure 7).

## Discussion

Gout is a form of inflammatory arthritis caused by formation of MSU crystals in the synovial tissues of joints [1]. IL-1β has been identified as a pivotal cytokine in gout and MSU crystal-induced inflammation [25]. Recent studies suggested that MSU-mediated NLRP3 inflammasome activation and subsequent IL-1β production in macrophages as key events in initiation of gout [2]. The aim of this study was to determine whether MSU-mediated inflammasome activation could be induced in non-myeloid synovial fibroblasts. A variety of structurally diverse molecules, including ATP, bacterial toxins, crystals, and amyloid proteins, are known to activate the NLRP3 inflammasome leading to IL-1β secretion [26]. Here we found that SAA, which is endogenously induced as an acute phase reactant, sensed the MSU-mediated caspase-1 activation and pro-IL-1β processing. The NLRP3 inflammasome pathway should be pivotal in this SAA/MSU-mediated IL-1β induction, since silencing NLRP3 using siRNA resulted in the abortive IL-1β induction. These data implicate a casual role of SAA in the pathogenesis of MSU-mediated inflammasome activation as well as acute inflammation seen in gouty arthritis.

IL-1β requires cleavage *via *caspase-1 for proper secretion, which is facilitated as a consequence of inflammasome assembly and activation [26]. The NLRP3 inflammasome has emerged as a critical sensor for a number of endogenous mediators, including MSU, that are capable of promoting IL-1β secretion [25]. However, our study demonstrated that MSU alone did not induce caspase-1 activation or IL-1β secretion in human synovial fibroblasts. Because of its pro-IL-1β inducing effect, SAA-priming of synovial fibroblasts could be essential for MSU-induced IL-1β secretion. Our data also suggest that SAA-induced *NLRP3 *mRNA expression and cathepsin B secretion may contribute to MSU-mediated NLRP3 activation.

Several lines of evidence indicate that toll-like receptor (TLR) ligands can elicit inflammasome activation [27]. Our findings suggest that SAA, a non-bacterial endogenous product, is sufficient to trigger caspase-1 activation and IL-1β processing in response to MSU, providing a mechanism for activation of the NLRP3 inflammasome in human synovial tissues. Endogenous molecules may be the first signal to prime the activation of the NLRP3 inflammasome, resulting in cooperative signaling [28]. The second signal is provided by stimuli that specifically activate NLRP3 and leads to caspase 1 activation and IL-1β processing [28]. Our results suggest that an endogenous proinflammatory molecule, SAA, could be the first signal to prime the activation of the NLRP3 inflammasome.

## Conclusion

Our data indicate that SAA induced MSU-mediated NLRP3 inflammasome activation and post-translational processing of IL-1β in human synovial fibroblasts. These findings highlight the potential role of SAA, a highly sensitive acute phase reactant, in the triggering of MSU-mediated acute synovial inflammation. The innate immune systems, including TLRs, are thought to be essentially involved in inflammasome-mediated inflammation [27]. However, our data show that interaction of an endogenous and non-bacterial acute phase protein, SAA, and MSU crystals synergistically enhance the inflammatory response by activating the inflammasome pathway. These findings provide a new insight into the mechanisms underlying acute gout.

## Abbreviations

ATP: adenosine triphosphate; ASC: apoptosis-associated speck-like protein containing CARD; IL-1β: interleukin-1β; NLRP3: Nod-like receptor family: pyrin domain containing 3; MSU: monosodium urate; SAA: serum amyloid A; TLR: toll-like receptor.

## Competing interests

The authors declare that they have no competing interests.

## Authors' contributions

KM, AK, TT, KS, MI, YM and YJ carried out cell culture and biochemical analysis. TK, YI, TM and MN participated in the design of the study and performed the statistical analysis. AK, SM, HI and TN conceived the study, participated in its design and coordination, and helped to draft the manuscript. All authors read and approved the final manuscript.
